# Enhancing Educational Programs and Communication Strategies to Address Alcohol Use Among EMS Providers

**DOI:** 10.66815/ajqr/19659

**Published:** 2026

**Authors:** Maria DH Koeppel, Beatrice Shlansky, Sara A Jahnke

**Affiliations:** National Development and Research Institutes, Leawood, KS, USA; Independent Researcher, Philadelphia, PA, USA; National Development and Research Institutes, Leawood, KS, USA

**Keywords:** Alcohol, Education, Emergency Medical Service Providers

## Abstract

Emergency Medical Services (EMS) providers experience substantial occupational stress, which may contribute to alcohol use as a coping mechanism. Despite growing attention to workforce wellness among first responders, relatively little research has examined how alcohol-related education should be developed and delivered for EMS providers. This qualitative study explored non–fire-based EMS providers’ perspectives on alcohol-related education, training, and support through semi-structured interviews with 31 EMS clinicians representing multiple certification levels and regions of the United States. Participants consistently described existing alcohol-related education as limited, passive, and focused primarily on patient care rather than provider well-being. They recommended educational approaches that incorporate EMS-specific data, information on the physiological effects of alcohol, personal narratives, and interactive instructional strategies. Participants also emphasized the importance of broader support systems, including peer support, family involvement, and improved awareness of available resources. Taken together, these findings suggest that alcohol-related education in EMS should be tailored to the occupational realities of the profession and integrated with broader efforts to support workforce health and well-being.

Emergency medical services (EMS) providers work in demanding environments characterized by frequent exposure to trauma, irregular schedules, sleep disruption, and chronic occupational stress. Occupational stressors such as role overload and ambiguity have been associated with anger and psychological distress (Doyle et al., 2021). In EMS, these individual-level stressors often occur alongside staffing shortages, inconsistent leadership, limited supervisor support, shift work, and other challenging workplace conditions that may contribute to mental health symptoms (Barth et al., n.d.; Edgelow et al., 2022). Together, these occupational demands have been linked to elevated rates of mental and physical health concerns among EMS personnel, including depression, sleep disorders, post-traumatic stress disorder (PTSD), and cardiovascular disease (Cash et al., 2021; Hegg-Deloye et al., 2014).

As concern for workforce wellness has grown, increasing attention has been directed toward the coping strategies used to manage chronic occupational stress and trauma exposure. Despite this interest, relatively little research has focused specifically on alcohol use among EMS providers. EMS providers represent a large but comparatively understudied segment of the first responder workforce. In the United States, EMS personnel respond to millions of emergency calls annually and serve as a critical link between public safety and the health care system. Yet, compared with police and fire service populations, limited research has examined the prevalence, patterns, and consequences of alcohol use among EMS providers. What research is available about EMS clinicians is often focused on fire-based EMS providers who are embedded within fire departments. Little attention has been paid to non-fire-based clinicians who operate in a very different environment.

This gap is important because alcohol use may exacerbate many of the health risks already prevalent in EMS. Existing first responder research may also not fully capture the experiences of EMS personnel because EMS systems vary considerably in organizational structure, including fire-based, hospital-based, private, volunteer, and other non–fire-based models. These organizational differences shape workplace culture, peer interactions, and social norms, suggesting that alcohol-related risks, attitudes, and educational needs may differ across EMS settings and from other first responder professions.

Unaddressed alcohol use among EMS providers may affect not only individual well-being but also occupational performance, patient care, and public safety. Alcohol use may worsen sleep disruption, impair recovery from stress, contribute to mental and physical health concerns, and affect readiness for duty. These risks are particularly relevant in EMS, where providers operate emergency vehicles, perform complex clinical interventions, and make critical decisions in high-stakes environments. Addressing alcohol use in this workforce therefore requires prevention and education strategies that recognize both the health needs of EMS personnel and the responsibilities of their professional role.

These concerns highlight the importance of understanding not only patterns of alcohol use among EMS providers but also how education and support initiatives can be used to address alcohol-related risks. Education and communication are potential organizational strategies to increase awareness, promote healthy behaviors, and facilitate access to support resources. EMS education traditionally emphasizes clinical knowledge and technical skills, with comparatively little attention devoted to self-care, stress management, resilience, or healthy coping strategies. As a result, EMS personnel may be well prepared to care for patients affected by excessive alcohol use but receive little formal education on how to recognize, prevent, or address alcohol-related concerns in themselves or their coworkers.

Occupational health literacy emphasizes that workers need both knowledge and organizational support to access, understand, and apply health information (Nutbeam, 2000). Organizations, therefore, play an important role in communicating health information and providing training and support systems that are accessible and actionable. Research on workplace health promotion, substance use prevention, and mental health literacy suggests that educational programs can improve knowledge, reduce stigma, and encourage help-seeking, particularly when training is interactive, tailored to the target audience, and reinforced by organizational support (Moll et al., 2018). Educational materials that are culturally relevant and personally meaningful are more likely to be accepted and translated into behavior change than generic informational approaches (Noar et al., 2007).

Understanding how EMS providers perceive existing alcohol-related education is an important step toward developing meaningful interventions. Although previous research provides general guidance on alcohol-related health promotion, direct input from EMS providers is needed to identify educational approaches relevant to the profession and responsive to its unique occupational demands. Qualitative methods are well-suited to exploring these perspectives because they allow providers to describe their experiences, educational needs, and recommendations in their own words. The present study leverages qualitative interviews with non–fire-based EMS providers to examine how alcohol-related education is currently delivered, identify perceived gaps in training and support, and explore recommendations for improving educational and organizational approaches to alcohol-related risks. By examining provider perspectives, this study seeks to inform the development of practical, EMS-specific strategies that support workforce health and well-being.

## Methods

### Data Source and Study Context

This manuscript draws on qualitative interview data collected as part of a larger National Institutes of Health funded study examining alcohol use among non-fire-based emergency medical service providers in the United States. The overarching project employed a mixed methods design to characterize alcohol use patterns, occupational risk factors, and health and workplace outcomes within the EMS workforce. The present analysis focuses exclusively on interview data collected to inform the development of education, communication, and organizational strategies addressing alcohol use in EMS settings.

The qualitative component was designed to capture EMS providers’ perspectives on how alcohol-related information is communicated within the profession, perceived gaps in education and messaging, and opportunities to improve workplace-based approaches to alcohol prevention and support. All study procedures were reviewed and approved by the Institutional Review Board (Approval #FWA00028794).

### Qualitative Design and Participant Selection

We conducted individual, semi-structured interviews with EMS providers using multiple convenience-based recruitment strategies. Study invitations were distributed through national EMS organizations, state level EMS offices across six states, and direct outreach to EMS agencies operating in multiple regions of the United States. Recruitment materials included a brief description of the study focus and instructions for expressing interest in participation. Eligible participants included currently practicing emergency medical technicians, advanced emergency medical technicians, and paramedics working in non-fire-based EMS systems. All interested providers who filled out the screener were invited to participate.

To capture a range of experiences relevant to alcohol education and communication, participants were screened and categorized based on self-described alcohol use as abstinent, moderate, or heavy. Abstainers were classified as those who had not consumed alcohol for at least the past 30 days, including those who have never consumed alcohol in their life. Moderate drinkers included those whose average daily intake (based on the number of drinks consumed each week) was not higher than 2 drinks per day for men or not higher than 1 drink per day for women. Heavy drinkers were defined as those who consumed alcohol at rates higher than those classified as moderate (National Institute on Alcohol Abuse and Alcoholism, 2026). Recruitment proceeded iteratively, with concurrent data collection and analysis guiding decisions about continued enrollment.

### Interview Procedures and Data Collection

Thirty-one interviews were conducted between February 2024 and October 2025 by research staff. All interviews took place via telephone using a secure line that allowed for digital audio recording. Interview duration typically ranged from 30 to 60 minutes. At the start of each interview, participants were provided with a standardized explanation of the study, including confidentiality protections and the voluntary nature of participation. Verbal informed consent was obtained prior to initiating the interview. Interviews followed a semi structured guide designed to elicit detailed discussion while allowing participants to introduce topics they viewed as salient. Interview questions explored experiences with alcohol related training and education, informal communication about alcohol use among peers, perceptions of organizational messaging and policies, and suggestions for improving education, awareness, and support within EMS agencies. Probing questions were used to clarify responses and explore examples offered by participants. Data collection and analysis occurred concurrently, allowing the research team to assess the emergence of new concepts throughout recruitment. As interviews progressed, the research team reviewed transcripts, compared emerging concepts across participants, and discussed whether additional interviews were contributing substantively new information relevant to the study aims. Saturation was determined when successive interviews reinforced previously identified concepts and no longer generated new themes or subthemes related to alcohol education, communication, and support.

### Analytic Approach

Interview recordings were transcribed verbatim and reviewed against the original audio files to ensure completeness and accuracy prior to analysis. The analytic process followed an inductive qualitative strategy informed by principles of grounded theory (Glaser et al., 1968; Miles & Huberman, 1994), with the goal of identifying patterns in perspectives, experiences, and organizational influences related to alcohol use among EMS providers.

Members of the research team independently read full transcripts to develop familiarity with the content and to note preliminary ideas and recurring concepts. These initial readings informed the development of a working coding structure, which was refined through iterative team discussion. Coding was conducted by two primary analysts who independently applied codes across the dataset. The coding framework evolved throughout the analytic process, with codes added, collapsed, or clarified as new patterns emerged. All coding decisions, code definitions, and thematic interpretations were developed and applied by the research team, maintaining a human driven analytic process.

Qualitative data management software (Dovetail) was used to organize transcripts, store coded excerpts, and facilitate retrieval of text segments associated with specific codes. The software functioned solely as a data management tool; it did not generate codes or interpret findings. Following completion of coding, generative AI tools (NotebookLM) were used in a limited and supportive capacity to assist with the organization of coded data and the identification of illustrative quotations within established thematic groupings. These tools were applied only after codes and themes had been defined by the research team and were not used to generate or modify analytic interpretations. All excerpts identified through these tools were cross checked with the original transcripts to confirm accuracy and contextual integrity.

To strengthen analytic rigor and credibility, two primary analysts independently coded the dataset and compared coded transcripts. Coding discrepancies and differences in interpretation were discussed and resolved through consensus. The coding framework was iteratively refined throughout analysis. Additional members of the research team reviewed coding outputs and thematic organization, providing an additional level of analytic review. Representative quotations were selected and verified against the original transcripts to confirm accuracy and contextual integrity.

### Research Reflexivity

The research team brought varied professional and research backgrounds to the study. The lead researcher has professional experience in EMS and currently serves as a firefighter and EMT, providing familiarity with EMS culture, terminology, and occupational experiences. The research team recognized that this experience could inform interpretation of participant accounts while also introducing assumptions based on prior occupational knowledge. To address this potential influence, coding and thematic interpretations were reviewed collaboratively, including by researchers without the same direct EMS experience, and differences in interpretation were discussed throughout the analytic process. This approach allowed the team to consider how researcher perspectives may have influenced the interpretation of the data while maintaining findings grounded in participant accounts.

### Results

As shown in Table 1, a total of 31 EMS providers participated in the interviews. Seventeen respondents reported working in large agencies (200+ providers), 7 in medium agencies (50–200 providers), 4 in small agencies (<50 providers). Three participants did not know their agency size. The average time in the field was 15.7 years, with time ranging from 1 to 47 years. Most participants were in the central United States, with 21 respondents from this region, compared to 6 from the west and 4 from the east. Over half of the participants were licensed as paramedics, while others identified as EMT B, EMT A, or another licensure category. With respect to alcohol use, 5 participants identified as abstainers, 19 as moderate drinkers, and 7 as heavy drinkers, as defined by the National Institute on Alcohol Abuse and Alcoholism (NIAAA) (National Institute on Alcohol Abuse and Alcoholism, 2026). The sample was predominantly male, with only 10 female participants. Most participants identified as non-Hispanic, and nearly all identified as White. The average age of participants was 41.6 years, with ages ranging from 20 to 72 years. While the EMS field tends to be predominately male and non-Hispanic white, this sample is somewhat older and less racially and ethnically diverse than the national population of EMS providers (Rivard et al., 2021).

Common themes and subthemes from the data are presented in Table 2. These findings focus on training and educational programs regarding alcohol use, strategies for program development, and concepts of support around high-risk alcohol use. Each of the four main themes includes a number of subthemes. These subthemes provide additional context on how the main theme exists in the current EMS climate. It should be noted that although the themes related to current training programs and pedagogical engagement strategies address overlapping aspects of education, they represent conceptually distinct findings. Current Training Programs captures participants’ experiences with existing alcohol-related education, whereas Pedagogical Engagement Strategies reflects participants’ recommendations for how future education should be delivered.

### Current Training Programs

Participants consistently described alcohol-related training and educational programs for EMS providers as minimal or entirely absent. Although EMS providers complete extensive annual continuing education, often totaling several hundred hours, little if any time is devoted to alcohol use, particularly as it relates to the health and wellness of the workforce. As one participant explained, “I can’t say that I’ve ever been required to sit down for a session or seen a session offered that did look inward at EMS at how to deal with alcoholism or excessive use of alcohol for us”.

When alcohol-related content is included, participants described it as a routine administrative requirement rather than a meaningful opportunity to address provider wellness. Training was commonly delivered through passive formats, such as online modules, which fostered a “check-the-box” mentality in which providers focused on completing the requirement rather than engaging with the material. One participant summarized this experience by stating, “‘Okay, yeah. Seen it, done it,’ click, click, click. I got 8,000 other things to get done”. Another noted, “Sometimes you can scroll straight through it without reading anything, click complete, and you get your credits”. Participants consistently described these instructional approaches as poorly aligned with the hands-on nature of EMS work. As one provider explained, “We don’t do well in lectures…We also like to be moving and doing things”. Another added, “I think if you just have ‘em sit there and watch PowerPoint slides, you’re gonna lose ‘em in the first two minutes.” Even when the topic itself was considered important, participants reported limited engagement with existing educational formats. As one participant stated, “Even just clicking through videos, or whatever education stuff, unless it’s engaging, we’re gonna get very bored easily”.

Participants also emphasized that the limited alcohol-related education they do receive is almost exclusively patient-oriented. Training typically focuses on recognizing and managing intoxicated patients as part of clinical education rather than addressing providers’ own alcohol use or helping them identify problematic drinking in themselves or their coworkers. One participant noted, “Aside from the basic sort of this is how a drunk person presents themselves as part of our medical license, no”, while another shared “I can’t say that I’ve ever been required to sit down for a session or seen a session offered that did look inward at EMS at how to deal with alcoholism or excessive use of alcohol for us”. Another participant summarized this distinction by explaining that alcohol was discussed in the context of “talking about it in the terms of a patient, yes. I think alcohol comes up as part of patient care, but not as far as taking care of each other or as a personnel thing”.

Overall, participants described current alcohol education for EMS providers limited, passively delivered, and focused primarily on patient care rather than provider health and wellness.

### Content for EMS Curriculum

Participants provided detailed recommendations regarding the content they believed should be included in an effective alcohol education curriculum for EMS providers. Across interviews, participants emphasized that educational materials must be relevant to the occupational culture and professional identity of EMS personnel. They described a curriculum that combines evidence-based information and scientific explanations of alcohol’s effects with personal narratives and case studies that increase relatability and emotional impact. Participants also emphasized the importance of helping providers recognize early warning signs of problematic alcohol use in themselves and their peers so that education serves a preventive rather than reactive function.

A frequently cited recommendation was to include objective, data-driven information. Participants indicated that EMS providers are more likely to engage with educational content when it includes hard statistics regarding alcohol use within the profession rather than generic health warnings or stereotypes. As one participant explained, “I know one thing that would resonate well with how a lot of first responders receive information, and it’s showing data”. Another participant emphasized the importance of using occupation-specific information, noting the need “to find facts and, not stereotypes, but statistics and facts of EMS, and fire, and usage, and stuff that can relate to us”. Participants viewed this type of evidence as essential for establishing credibility. As one provider stated, it was “critical because if you can’t really show ‘em numbers, solid numbers that are valid—it’s [just] another class that I’m required to take.”

In addition to prevalence data, participants expressed a strong preference for educational content that reflects their medical training. Many described wanting detailed explanations of the physiological effects of alcohol on the body, including its impact on specific organ systems, brain function, and long-term disease risk. One participant suggested including “more of the actual reaction, the actual effect it has on your heart, the actual effect it has on your kidneys and liver”. Others noted the importance of addressing broader health consequences, including “… the stress on your heart or just the cancer rates and things”, and “how it affects your brain specifically over time”. Participants also expressed interest in understanding alcohol metabolism and the time required for alcohol to clear the body. As one participant explained, “I think that it would be really cool to see study information on the effects of alcohol use and the hours it takes for the alcohol to completely get out of your system”. Participants noted that this information could also provide a scientific basis for agency “bottle to throttle” policies, which specify the minimum time allowed between alcohol consumption and reporting for duty.

Finally, participants emphasized the importance of pairing scientific information with personal narratives and case studies. Stories involving local colleagues or recognizable examples were viewed as more compelling than abstract data or accounts involving providers from distant locations. As one participant explained, “stories, or incidences that are local to us, that would be some information that we can resonate better with... If we have something close versus a medic in [another state]. We’re not gonna resonate with that at all”. Another participant noted that these examples help reinforce the relevance of the issue by demonstrating that “These are people in EMS that had these things happen to them. These are cases—these are friends of ours”. Participants described these narratives as transforming information into something personally meaningful. One provider stated that stories make “it all feel real versus just data, or information on a page”, while another emphasized “I can tell anybody any facts; as long as they don’t have an emotional context to that, they can’t see themselves within it; it doesn’t really do anything... You have to have that emotional connection”.

Taken together, participants described an ideal EMS-specific alcohol curriculum as one that combines scientific credibility with personal relevance. Objective data regarding alcohol use in EMS, detailed explanations of physiological effects, and emotionally resonant narratives were viewed as complementary components of an effective educational approach. By aligning content with the medical background and occupational culture of EMS providers, participants believed educational programs would be more likely to capture attention, foster self-reflection, and support behavior change.

### Pedagogical Engagement Strategies

Participants emphasized that effective alcohol education for EMS providers requires instructional approaches that move beyond traditional lecture-based formats. Given the experiential, hands-on nature of EMS work, participants consistently preferred interactive learning strategies that actively involve learners rather than passive presentations. In addition to experiential activities, participants highlighted the importance of relatable messaging and the strategic use of humor to create culturally appropriate, engaging educational experiences.

Participants frequently noted that EMS providers learn best through direct participation and practical demonstration. Traditional classroom lectures were widely described as ineffective because they often lead to boredom and disengagement. In contrast, participants recommended hands-on tools and simulations that allow providers to experience the effects of alcohol in a controlled setting. Drawing on examples from other substance use education programs, one participant suggested, “give us the drug goggles, give us the rangers”, explaining that “EMS providers, being that we’re a very cynical bunch... they truly just need to see for themselves how bad they really are, or how bad they can be at different thresholds”. Another participant described a television program in which individuals consumed alcohol, completed breathalyzer tests, and were video recorded while intoxicated. After sobering up, they watched the recordings and experienced “that moment of realization of oh my gosh, this is what I’m like?” Participants suggested that activities promoting this type of self-recognition could be particularly effective in overcoming denial and increasing awareness of impairment. In addition to simulations, some participants recommended self-assessment tools. As one provider explained, a “quick, self-survey type of deal, self-questionnaire, that was accurate, and it had the validity and reliability built behind it…might wake up some people”.

Participants also stressed that educational messages must be framed in ways that feel personally relevant and culturally authentic. Training that appears disconnected from the realities of EMS work was often dismissed as an administrative directive rather than a meaningful wellness initiative. As one participant noted, “if you’re able to make something we can actually relate to, great, wonderful. Otherwise, it just comes across as a management move, and we’re like, ‘Nah, another management move, whatever’”. Participants suggested that messaging should encourage providers to reflect on their own behavior by drawing on the same concern they would show toward a coworker. One participant explained that “a lot of it is just reminding people to look within themselves. Just the same way that you would say if you had a friend who—not a patient. If you had a friend who was doing this, what would you say?” Another participant summarized the importance of personalization by stating that “it really is about personalization of how this impacts you directly and what does this mean to your future as an EMS provider”.

A final strategy identified by participants was the use of humor. Humor was described as a deeply embedded feature of first responder culture and a familiar way of coping with occupational stress. Participants believed that incorporating humor into educational materials could make discussions of alcohol use and wellness more approachable and reduce resistance to the content. As one participant explained, humor is important “because I know people might feel maybe they’re being beat over the head with it [alcohol education]”. Another noted that, without this approach, training can feel like providers are “almost being back in middle school and talking about drugs and drinking and all of that”. Participants viewed humor as a way to maintain engagement, foster camaraderie, and present serious topics in a manner consistent with the culture of EMS.

Overall, participants described effective pedagogical strategies as those that are interactive, relatable, and culturally aligned with the EMS profession. Hands-on activities, personalized messaging, and the thoughtful use of humor were viewed as essential for transforming alcohol education from a routine administrative requirement into an engaging and meaningful learning experience.

### Integrated Support

Participants described effective alcohol education as extending beyond the delivery of information to include a broader support framework that addresses problematic alcohol use before it escalates into a crisis. This framework was characterized as proactive and multi-layered, involving individual providers, peers, family members, and organizational resources. Key components included education on early warning signs, peer-led intervention, family involvement, and improved awareness of available support services.

Participants emphasized that recognizing early warning signs of problematic alcohol use is a foundational element of prevention. They described behavioral and emotional changes such as irritability, sleep disturbances, nightmares, increased defensiveness, and morning drinking as indicators that a provider may be struggling. One participant explained the importance of helping providers and families “know about the subtle signs to look for. If we start to see a change in somebody off duty and, you know, their drinking habits, or their personality, or their defensiveness...”. Another participant reflected on personal experience, stating, “I didn’t sleep, and then I got irritated, and I got angry at people.” Participants also noted that coworkers often observe physical signs of impairment, asking questions such as, “How’s your partner acting today? Do they seem like they’re struggling to drive or struggling to walk.” As one participant summarized, “Alcoholism is a problem in EMS... We’re going to talk about the warning signs”. These observations suggest that EMS providers already recognize many indicators of problematic alcohol use, even when they lack formal language or training to interpret them.

Peer-led intervention emerged as one of the most consistently endorsed strategies for addressing alcohol-related concerns. Participants emphasized that coworkers are often best positioned to identify behavioral changes and initiate supportive conversations because they understand the profession’s unique demands. One participant noted that interventions may feel more authentic “if it’s your own peers... it’s almost seen as more we care about you more than it would management coming across”. Participants also described peer support as a form of informal accountability rooted in the interdependence of EMS work. As one provider explained, “Nobody wants to partner with someone who’s impaired because we depend on each other”. This shared responsibility can facilitate direct and supportive action, as illustrated by one participant who described saying, “all right, hey buddy, I realize this is what’s going on... You need to call in sick and go home now and take steps to figure out why you’re in this place”. Participants stressed that confidentiality is essential to the success of peer-based approaches. As one provider stated, “Honestly, my first step would probably be go to the coworker and let them know that I’m gonna let peer support know, which is completely confidential, and that management would never find out”.

Participants also identified spouses and family members as important contributors to early detection and support. Because EMS providers may conceal problems in the workplace, family members are often the first to notice changes in mood, behavior, and drinking patterns. One participant observed that “our spouses are—you know, are going to be the ones that see the subtle changes”, while another emphasized the importance of “families involved, because those are the people that are close to us that notice our—the changes in us”. Participants noted that this role is particularly important because “I think we are able to hide a lot. We have a super hard shell”. Family involvement was also viewed as relevant because work-related stress and time away from home can contribute to interpersonal strain, which may in turn increase alcohol use. As one participant explained, “We’re away from our families a lot. Obviously, there can be some tension there. ... I can see where being at work, or even drinking when they’re not at work might lead to more issues, or might lead them to drink more”.

Finally, participants emphasized the importance of improving resource literacy. Although many providers were generally aware that resources such as Employee Assistance Programs (EAPs) existed, they often lacked specific knowledge about how to access them or whether they were truly confidential. One participant noted, “It’s not like someone comes around and hands out pamphlets or someone comes and talks to us, but it’s known”, while another stated “I know there are resources. I’m just not exactly sure. ... Trying to think. There’s an employee assistance program…”. Participants also described skepticism regarding the relevance of these services, often minimizing their own drinking by comparing themselves to others. As one participant explained, “I don’t believe most EMS providers... would actually use them because of that thing that we talked about before about I’m really not that bad, or my situation isn’t as worse as…”. Another participant linked this reluctance to the professional identity of EMS providers, noting that many avoid seeking help because “they don’t wanna admit that they are now the patient. They don’t wanna be the patient…”. Participants suggested that educational programs should include concrete information on how to access services, reinforce confidentiality protections, and provide examples demonstrating that support resources are both effective and relevant. As one participant summarized, “Having it localized to support whatever evidence in teaching will actually I think make an impact to most people.”

Participants described integrated support as an essential complement to alcohol education. Training that helps providers identify early warning signs, equips peers and families to intervene, and increases awareness of confidential support resources was viewed as critical to creating a preventive culture in which alcohol-related concerns can be recognized and addressed before they result in significant personal or occupational consequences.

The findings presented in this results section highlight a clear disconnect between the role alcohol appears to play in EMS culture and the limited educational and organizational responses currently available to address it. Participants consistently described existing training as minimal, passive, and focused on patient care rather than the health and wellness of EMS providers themselves. At the same time, they articulated a detailed, highly specific vision of what effective alcohol education should include. This vision emphasized scientifically grounded, occupation-specific content; engaging instructional strategies tailored to the experiential learning preferences of EMS providers; and an integrated support system involving peers, families, and confidential professional resources. Taken together, these themes suggest that alcohol education in EMS cannot be approached as a generic workplace wellness topic. Rather, participants’ perspectives suggest that future programs should be culturally relevant, medically credible, and responsive to the unique social and occupational realities of EMS work.

## Discussion

This study explored non–fire-based EMS providers’ perspectives on alcohol-related education and support within the EMS workforce. Participants consistently described existing alcohol-related education as limited, passive, and focused primarily on patient care rather than provider well-being. At the same time, they provided detailed recommendations for improving educational efforts, including the use of EMS-specific content, interactive instructional strategies, and greater emphasis on peer, family, and organizational support. Taken together, these findings suggest that alcohol-related education in EMS should extend beyond information delivery to encompass broader approaches that promote awareness, early risk identification, and access to resources.

Participants consistently emphasized the importance of EMS-specific content when discussing alcohol-related education. Generic training programs were often described as easy to dismiss, whereas occupation-specific statistics and scientifically grounded information were viewed as more credible and relevant (Kreuter & Wray, 2003). Participants expressed a strong preference for educational content that reflected their medical training, including information on the physiological effects of alcohol, long-term health consequences, and alcohol metabolism. They also emphasized the importance of using data specific to EMS, suggesting that educational efforts may be more effective when providers can see how alcohol use affects their own profession rather than the general population.

Participants also highlighted the importance of instructional format. Passive online modules and lecture-based approaches were frequently described as unengaging and ineffective. In contrast, participants recommended interactive learning strategies, including simulations, self-assessments, and discussion-based activities. These preferences likely reflect the experiential nature of EMS education, where hands-on learning is commonly used to develop clinical knowledge and skills. Together, these findings suggest that both content and delivery format should be considered when developing alcohol-related educational programs for EMS providers.

Participants also emphasized the importance of personal narratives and emotional relevance. Many described stories involving coworkers, local providers, or recognizable EMS personnel as more engaging than factual information alone. Participants suggested that these narratives make alcohol-related information more relevant by connecting educational content to real-world experiences within the profession. Similarly, humor was viewed as a useful strategy to increase engagement and present a sensitive topic in a manner consistent with EMS culture.

These findings are consistent with previous research suggesting that educational messages are more likely to be accepted when they are perceived as personally relevant and culturally appropriate. Educational approaches that incorporate peer experiences and real-world examples may therefore represent a useful complement to more traditional information-based instruction (Winograd et al., 2024). Future research should examine whether these strategies improve engagement, knowledge retention, or willingness to seek support among EMS providers.

Participants did not view education as sufficient on its own. Rather, they described alcohol-related prevention as requiring a broader support system that includes peers, family members, and organizational resources. Participants frequently identified coworkers as important sources of support because they understand the unique demands of EMS work and are often in the best position to recognize changes in behavior, initiate conversations, and encourage help-seeking. Many participants emphasized that these interactions are most effective when they occur in a supportive and confidential environment.

Family members were also viewed as important contributors to early recognition and intervention. Participants noted that spouses and other family members may observe changes in mood, behavior, and drinking patterns that are not readily apparent in the workplace. Given the amount of time EMS providers spend away from work, participants suggested that family members may play an important role in recognizing concerns and encouraging providers to seek assistance when needed.

Taken together, these findings suggest that alcohol-related education may be most effective when paired with support structures that help providers recognize concerns, access resources, and engage in supportive conversations before alcohol-related problems escalate. Participants emphasized the importance of combining educational content with information on warning signs of problematic alcohol use, available support resources, and strategies for helping peers seek assistance. Educational initiatives may therefore benefit from integration with broader organizational efforts that promote awareness, reduce stigma, and facilitate access to support.

These findings have several practical implications for EMS agencies and educators. Importantly, these implications reflect participants’ perceptions of potentially useful educational approaches rather than evidence of intervention effectiveness. Based on participant recommendations, future curricula could incorporate EMS-specific prevalence data, information on the physiological effects of alcohol, early warning signs of problematic use, and guidance on accessing confidential support resources. Participants also expressed a preference for interactive instructional approaches, including case-based learning, simulations, self-assessments, and discussion-based activities. These findings therefore provide a preliminary, provider-informed framework for developing alcohol-related educational programs that can be evaluated in future research.

Several limitations should be considered when interpreting these findings. Participants were predominantly White and somewhat older than the national EMS workforce, which may limit the transferability of findings to younger providers and more diverse populations. In addition, this study focused exclusively on non–fire-based EMS providers. Although this focus addresses an important gap in the literature, educational needs and organizational approaches may differ in fire-based, volunteer, hospital-based, and other EMS delivery models. Participation was also voluntary and relied on convenience-based recruitment, creating the potential for self-selection bias. EMS providers who chose to participate may have differed from those who did not participate, including in their experiences with alcohol, interest in workforce wellness, or willingness to discuss alcohol-related concerns. As a result, the perspectives represented in these interviews may not reflect those of EMS providers who were less willing to engage with this topic. Finally, this study explored provider perspectives on alcohol-related education and support but did not evaluate the effectiveness of any specific intervention. Future research should build upon these findings by developing and testing EMS-specific educational programs and examining their impact on outcomes such as knowledge, attitudes, help-seeking, peer intervention, and alcohol-related risk behaviors.

Alcohol use remains an understudied aspect of EMS workforce health despite its potential implications for provider well-being, occupational performance, and workforce health. Participants in this study described clear gaps in current educational approaches and offered practical recommendations for improvement. Their perspectives suggest that effective alcohol-related education should be tailored to the realities of EMS practice, incorporate engaging and relevant instructional strategies, and be supported by organizational resources that encourage early recognition and help-seeking. By incorporating provider perspectives into program development, EMS agencies may be better positioned to create educational initiatives that are relevant, credible, and responsive to the realities of EMS practice.

## Figures and Tables

**Table 1:** Participant Characteristics

Size of Agency	N (mean)	% (range)

Large	17	54.8%
Medium	7	22.6%
Small	4	12.9%
Unsure	3	9.7%
Time in EMS (years)	15.7	1 – 47

**Region**		
East	4	12.9%
Central	21	67.7%
West	6	19.4%

**Licensure level**		
EMT B	8	25.8%
EMT A	1	3.2%
Paramedic	17	54.8%
Other	5	16.1%

**Drinking**		
Abstainer	5	16.1%
Moderate	19	61.3%
Heavy	7	22.6%

**Sex**		
Female	10	32.3%
Male	21	67.7%

**Ethnicity**		
Hispanic	2	6.5%
Non-Hispanic	29	93.5%

**Race**		
White	30	96.8%
Asian	1	3.2%
Black	0	0.0%

**Age (years)**	**41.6**	**20 – 72**

**Table 2:** Thematic Summary

Main Themes	Sub-Themes

Current training programs	- Passive instructional models - Patient-oriented
Content for EMS curriculum	- Evidence-based data - Physiological impact - Personal narratives/case studies
Pedagogical engagement strategies	- Interactive learning - Relatable messaging - Humor
Integrated support	- Early warning signs - Peer-led intervention - Resource literacy - Familial involvement

## Data Availability

The data that support the findings of this study are available on request from the corresponding author.
